# Cerebroventricular Microinjections of MPTP on Adult Zebrafish Induces Dopaminergic Neuronal Death, Mitochondrial Fragmentation, and Sensorimotor Impairments

**DOI:** 10.3389/fnins.2021.718244

**Published:** 2021-08-27

**Authors:** Michael Kalyn, Marc Ekker

**Affiliations:** Department of Biology, Faculty of Science, University of Ottawa, Ottawa, ON, Canada

**Keywords:** Parkinson’s disease, zebrafish, regeneration, dopamine neuron, neurotoxin, drug administration

## Abstract

Mitochondria are dynamic organelles that mediate the energetic supply to cells and mitigate oxidative stress through the intricate balance of fission and fusion. Mitochondrial dysfunction is a prominent feature within Parkinson disease (PD) etiologies. To date, there have been conflicting studies of neurotoxin impact on dopaminergic cell death, mitochondrial function and behavioral impairment using adult zebrafish. Here, we performed cerebroventricular microinjections (CVMIs) of 1-methyl-4-phenyl-1,2,3,6-tetrahydropyridine (MPTP) on adult transgenic zebrafish that resulted in significant reductions in dopaminergic neurons within the telencephalon and olfactory bulbs (OB) of Tg(*dat:eGFP*) fish. Visualization of mCherry and mitochondrial gene expression analysis in Tg(*dat:tom20 MLS:mCherry*) fish reveal that MPTP induces mitochondrial fragmentation in dopaminergic neurons and the activation of the pink1/parkin pathway involved mitophagy. Moreover, the loss of dopaminergic neurons translated into a transient locomotor and olfactory phenotype. Taken together, these data can contribute to a better understanding of the mitochondrial impact on dopaminergic survivability.

## Introduction

Idiopathic in nature, Parkinson disease (PD) is the second most prevalent neurodegenerative disease in the world where symptoms comprise of motor, sensory and cognitive impairments (de Lau and Breteler, 2006; Muangpaisan et al., 2011). The pathology of PD has been characterized as a progressive loss of dopaminergic neurons within the substantia nigra of the human midbrain (Dauer and Przedborski, 2003; de Lau and Breteler, 2006; Muangpaisan et al., 2011). Motor and sensory symptoms develop following 40–60% loss of these neurons (Cheng et al., 2010).

Parkinson disease is believed to be a cumulative result of genetic and environmental insult. Researchers have elucidated mutations in various genes [*PINK1* (Hatano et al., 2004), *Parkin* (Kitada et al., 1998; Dawson and Dawson, 2010), *LRRK2* (Funayama et al., 2002; Zimprich et al., 2004), and *DJ*-1(Bonifati et al., 2003)] that increase an individual’s susceptibility to developing PD. However, this can only explain 5–10% of cases, while the majority are sporadically induced through external factors such as: age, gender, neurotoxic exposure, and mitochondrial dysfunction (Dexter and Jenner, 2013).

Chemo-ablative models for PD commonly consist of the administration or exposure to 1-methyl-4-phenyl-1,2,3,6-tetrahydropyridine (MPTP; Meredith and Rademacher, 2011; Tieu, 2011; Hare et al., 2013). Being lipophilic, MPTP readily permeates the blood-brain-barrier and is metabolized into its active toxic form MPP+ through monoamine oxidase-B (MAO-B) in proximal astrocytes (Przedborski et al., 2004; Hare et al., 2013). MPP+ enters dopaminergic neurons through the dopamine transporter (Dat) protein to exert its cytotoxicity through the inhibition of complex I activity in the electron transport chain (ETC) of mitochondria (Przedborski et al., 2004). MPTP has been extensively used in PD studies in a variety of models ranging from mice, rats, primates, and zebrafish (Van Kampen et al., 2000; Meredith and Rademacher, 2011; Bové and Perier, 2012). Previous studies in zebrafish have been carried out using embryonic or larval stages in development. These studies have demonstrated the effect of MPTP on locomotion, morphology, dopaminergic survivability and have identified various neuroprotective compounds (Lam et al., 2005; Sallinen et al., 2009; Díaz-Casado et al., 2016; Kalyn et al., 2019; Zhao et al., 2020). However, studies pertaining to MPTP exposure in adult zebrafish address the behavioral phenotype but commonly disregard or report inconsistencies surrounding the impact on sensory perception, dopaminergic neurons and their mitochondria (Anichtchik et al., 2004; Bretaud et al., 2004).

Many studies have adopted the use of mammalian models to study PD, however, the use of zebrafish has rapidly emerged and proven to be considerably beneficial. Zebrafish possess transparent *ex utero* embryogenesis allowing for in-depth developmental imaging, as well as, large clutch sizes following breeding to facilitate high-throughput drug screening (Stewart et al., 2014). This model also displays many conserved pathologies and molecular pathways due to their genome being ∼87% homologous to humans (Du et al., 2016). Pertinent to PD, zebrafish possess a functional analogue to the dopaminergic nigrostriatal system described in humans (Holzschuh et al., 2001; Sallinen et al., 2009).

Given the accessibility of zebrafish embryos into adulthood, this model is widely used for pharmacological studies. Conventional drug delivery methods comprise of direct immersion, intraperitoneal (IP) injections and intramuscular (IM) injections, however, there are significant pitfalls to each. In this study, we optimized a drug delivery method through cerebroventricular microinjections (CVMIs) to administer MPTP to adult zebrafish. MPTP is shown to selectively ablate dopaminergic populations through mitophagy mediated cell death demonstrated through increased mitochondrial fragmentation, as observed in adult Tg(*dat:tom20 MLS:mCherry*) zebrafish. This effect was complimented with gene expression analyses of mitochondrial regulatory genes. Moreover, this decrease in dopaminergic neurons translates into transient sensorimotor perturbing phenotypes.

## Materials and Methods

### Zebrafish Care and Husbandry

The transgenic zebrafish lines used for this study are Tg(*dat:eGFP*) and Tg(*dat:tom20 MLS:mCherry*) (Xi et al., 2011; Noble et al., 2015). Embryos were collected by natural spawning to be raised in E3 embryo media (13 mM NaCl, 0.5 mM KCL, 0.02 mM Na_2_HPO_4_, 0.04 mM KH_2_PO_4_, 1.3 mM CaCl2, 1 mM MgSO_4_, and 4.2 mM NaHCO_3_). Zebrafish were sorted by fluorescent intensity detected at 4 dpf and raised into adulthood for experimentation. All procedures were approved by the University of Ottawa Animal care committee and conducted under the Animal Care and Veterinary Service guidance in accordance with the Canadian council for Animal care following protocol number BL-2081.

### 1-Methyl-4-Phenyl-1,2,3,6-Tetrahydropyridine Preparation and CVMIs in Adult Zebrafish

1-Methyl-4-phenyl-1,2,3,6-tetrahydropyridine (C_12_H_15_N⋅HCl, Product: M0896, CAS: 23007-85-4, Sigma, Oakville, ON, Canada) was reconstituted in distilled water to a stock concentration of 500 mM and diluted to the working concentrations of 10, 25, 35, and 100 mM.

Adult Tg(*dat:eGFP*) and Tg(*dat:tom20 MLS:mCherry*) zebrafish (∼10-month-old) were anesthetized with Tricaine. In accordance with the procedure outlined in Kizil et al., a small incision of 200 um was then generated with a 30-gauge syringe (BD Ultra-fine II, BD Biosciences) in the cranium above the anterior portion of the optic tectum without damaging the brain. The prepared MPTP was loaded into a thin glass capillary microinjecting needle (Sutter Instrument) to inject the contents through the incision into the cerebroventricular fluid surrounding the brain. Microinjections were performed using the IM-300 vacuum pump microinjector station (Narishige) at a pressure range of 55–65 psi. Phenol Red (Sigma) was co-administered to ensure proper distribution throughout the cerebroventricular fluid (CVF).

### Immunohistochemistry on Zebrafish Cryosections

Zebrafish heads were collected and fixed in a 4% paraformaldehyde (PFA) solution dissolved in 1× phosphate buffered saline (PBS) overnight at 4°C. Brain-dissections were conducted in 1× PBS and washed thrice in PBS-T followed by equilibration in 30% sucrose/PBS overnight at 4°C. The brains were flash frozen in Tissue-tek OCT (VWR) for coronal cryosectioning. Section thickness performed range from 16 to 20 μm and were stored at −20°C.

Sections were thawed for 30 min prior to respective antigen retrieval. Sections were placed in 0.05% sodium citrate in 1× PBS-T for 15 min at 85°C then cooled at RT for 15 min. Slides were then washed with 1× PBS twice prior to rehydration in 1× PBS-T for 10 min. Blocking was then performed, and the slides were incubated with primary antibodies diluted in 1% fetal bovine serum (FBS) in PBS-T overnight at 4°C. Primary antibodies used were; anti-GFP (polyclonal rabbit IgG, A11122, Invitrogen) and anti-DsRed (polyclonal rabbit IgG, #632496, Clontech). Slides were washed and incubated with the secondary antibodies for 2 h at RT in a dark chamber. Secondary antibodies used were; goat anti-rabbit Alexa 488 conjugate (A11008, Invitrogen) and goat anti-rabbit Alexa 594 conjugate (A11012, Invitrogen). Slides were then washed in 1× PBS-T prior to mounting with DAPI-infused Vectashield mounting media (Lynx Biosciences). Images for eGFP+ quantification were captured with the Olympus FV1000 Confocal microscope using a 20× air immersion objective and images for mCherry+ quantification were captured using a Zeiss LSM 880 Confocal microscope using a 63× oil immersion objective. Mitochondria were ranked according to their morphology. Fused refers to uniformly linked mitochondria surrounding the nucleus. Partial refers to mitochondrial fragmentations observed in less than 50% of the mitochondria within the field of view. Fragmented refers mitochondria that appear scattered (>50%), truncated and less dense surrounding the nucleus. Counts were performed using ImageJ software and were performed by two to three independent researchers in a blinded approach to remove personal bias.

### Whole Brain Real-Time Quantitative Reverse Transcription PCR (qRT-PCR)

RNA was extracted from homogenized adult whole zebrafish brains using TRIzol in accordance to the manufacturer’s protocol (Invitrogen, Thermo Fisher Scientific, Waltham, MA, United States). The purity and integrity of RNA was determined using the NanoDrop 1000 spectrophotometer (Thermo Fisher Scientific, Waltham, MA, United States) and through gel electrophoresis. Samples only with absorbance of 1.8–2.1 and clear 18S and 28S bands were used for cDNA synthesis. The reverse-transcription was performed using the iScript cDNA Synthesis Kit following the supplier’s protocol. qRT-PCR reactions were done in triplicates composed of 5 μL SsoFast^TM^ EvaGreen^®^ Supermix (Bio-Rad), 0.4 μL forward primer, 0.4 μL reverse primer, 0.2 μL nuclease-free water, and 4 μL cDNA. All readings were analyzed using the Bio-Rad CFX96 instrument. Relative quantification for *th1*, *dat*, *p53*, *pink1*, *parkin, mao-b, fis1*, *mfn1*, *opa1*, *parla*, *sox2*, and *nestin* transcripts was obtained through the comparative Cq method using the following reference genes; *ribosomal protein l13a* (*rpl13a*), *elongation factor 1 alpha* (*ef1a*), and *tyrosine 3-monooxygenase/tryptophan 5-monooxygenase activation protein*, *zeta polypeptide* (*ywhaz*). Oligonucleotide primer sequences are listed in Table 1.

**TABLE 1:** List of primers designed for qRT-PCR.

Primer	Forward Sequence (5′-3′)	Reverse Sequence (5′-3′)	References
*ywhaz*	TCTGCAATGATGTGTTGGAGC	TCAATGGTTGCTTTCTTGTCGTC	Tang et al., 2007
*rpl13a*	TCTGGAGGACTGTAAGAGGTATGC	AGACGCACAATCTTGAGAGCAG	
*ef1a*	CTGGAGGCCAGCTCAAACAT	ATCAAGAAGAGTAGTACCGCTAGCATTAC	
*Dat*	AGACATCTGGGAAGGTGGTG	ACCTGAGCATCATACAGGCG	Barreto-Valer et al., 2012
*th1*	GACGGAAGATGATCGGAGACA	CCGCCATGTTCCGATTTCT	Chen et al., 2016
*p53*	ATATCCTGGCGAACATTTGG	ACGTCCACCACCACCATTTGAAC	
*sox2*	GACAGCTACGCGCATATGAA	AGCCGTTCATGTAGGTCTGC	
*Nestin*	ATGCTGGAGAAACATGCCATTGCAG	AGGGTGTTTACTTGGGCCTGAAGA	
*fis1*	CCCTGAACCTTCCAGTGTTT	GTCTCTGGAAACGGGTCCTT	
*opa1*	GCTTGAGCGCTTGGAAAAGGAA	TGGCAGGTGATCTTGAGTGTTGT	
*mfn1*	CTGGGTCCCGTCAACGCCAA	ACTGAACCACCGCTGGGGCT	
*pink1*	GGCAATGAAGATGATGTGGAAC	GGTCGGCAGGACATCAGGA	
*mao-b*	CGTACATTGGACCAACTCAAAA	CCTCCAGAGGTTGTTGTAGTCC	Sarath Babu et al., 2016
*parkin*	GCGAGTGTGTCTGAGCTGAA	CACACTGGAACACCAGCACT	

### Locomotion Analysis

Following treatment and recovery, adult zebrafish were monitored for swimming activity. Fish were placed individually in static tanks and allowed to acclimate in the ambient light for 15 min prior to the trials. Recordings were taken using the ZebraCube tracking system (ViewPoint Life Sciences, Lyon, France) and analyzed with Zebralab software. The swimming parameters examined in this study consist of total distance traveled, average swimming velocity and freezing bout duration over the course of 7 min trials. Sample sizes of 10 were selected to delineate the effects of individual swimming variability. Additionally, the same fish were analyzed from the last injection and the recovery period.

### Olfactory Analysis

Following treatment and recovery, adult zebrafish were placed in a customized static tank that is comprised of a midline division to result in a neutral zone, left and right arm (Hussain et al., 2013). Fish were allowed 15 min for tank acclimation in the ambient ZebraCube lighting. Following acclimation, cadaverine (Sigma Aldrich) was added to the arm the fish was in at that time-point referred to as the stimulus arm. Trials of 3 min were conducted to limit cadaverine diffusion into the non-stimulus arm or neutral zone. Data was captured using the ZebraCube tracking system and interpreted with the Zebralab software.

### Statistical Analysis

All statistical analysis was performed using the software GraphPad Prism v7.0 (San Diego, CA, United States). Swimming parameters were examined for *n* = 10 adult zebrafish from each treatment group, while the olfactory evasion assay evaluated *n* = 5. Both data sets were analyzed using multiple *t*-test comparison with significance determined using the Holm-Sidak method. Gene expression analysis was collected from whole brains from *n* = 3 of each treatment group. Similarly, significance was quantified using multiple *t*-test and Holm-Sidak analysis. eGFP+ and mCherry+ cells, with *n* = 6 and 4, respectively, were analyzed using a two-way ANOVA with multiple comparisons. eGFP+ multiple comparisons were performed using the Sidak method, while mCherry + multiple comparisons used the Holm-Sidak method. Statistical significance was determined using a 95% confidence interval where *p* < 0.05. ^∗^*p* < 0.05, ^∗∗^*p* < 0.01, ^∗∗∗^*p* < 0.001, and ^****^p < 0.0001.

## Results

### Cerebroventricular Microinjection Optimization and the LC50 Dose for MPTP

**FIGURE 1:**
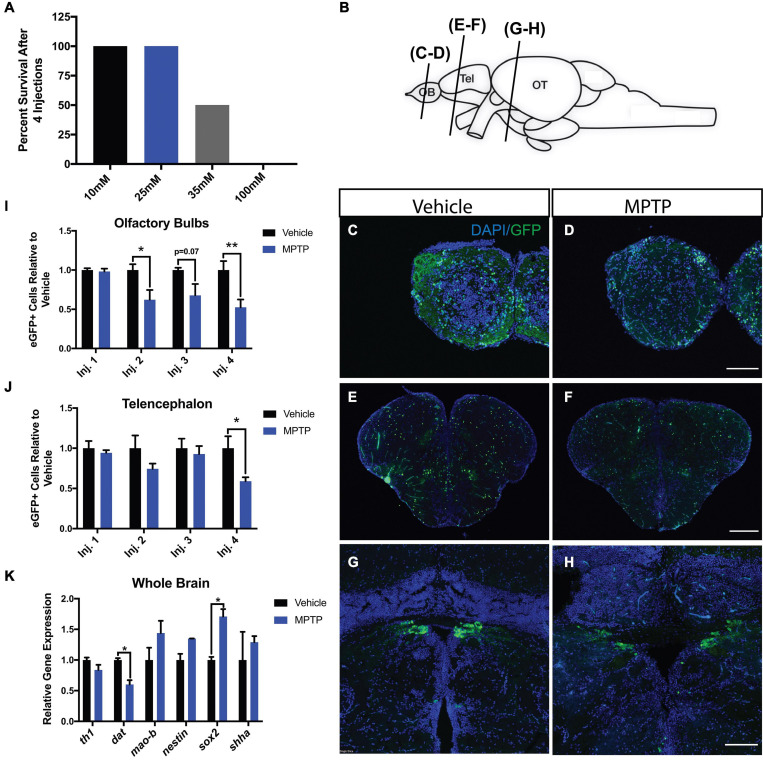
Impact of MPTP on various brain regions of adult Tg(*dat:eGFP*) zebrafish. **(A)** MPTP dose-response. **(B)** Schematic representation of the zebrafish brain with the planes of section indicated. Immunohistochemical labeling of eGFP (green) and DAPI (blue) to observe dopaminergic neurons in the olfactory bulbs (OBs) **(C,D)**, telencephalon **(E,F)**, and periventricular pretectal nucleus (PPv) **(G,H)**. **(I,J)** Quantification of eGFP+ cells within the OBs and telencephalon respectively. (*n* = 6). **(K)** Whole brain qRT-PCR analysis for dopaminergic, and regeneration-related genes; *th1*, *dat*, *sox2*, *nestin*, and *shha.* (*n* = 3 whole brains from each respective group). Bars represent the mean ± the SEM. *p < 0.05, **p < 0.01. Scale bars = 100 and 200 μm for the OB/PPv and telencephalon respectively. OB, olfactory bulbs; Tel, telencephalon; OT, optic tectum.

Cerebroventricular microinjection, based on the protocol of Kizil and Brand (2011), was performed on 10-month old adult Tg(*dat:eGFP*) and Tg(*dat:tom20 MLS:mCherry*) zebrafish. A range of MPTP concentrations was administered to 8 fish in each group to determine the sublethal dose. The range comprised of 10, 25, 35, and 100 mM concentrations of MPTP. Injections were performed daily for four consecutive days. Mortality rates were quantified following each injection point. A concentration of 100 mM was shown to be completely lethal following the last injection, whereas 35 mM resulted in 50% survival. Both 10 and 25 mM did not result in any observed death, thus 25 mM was determined to be the working dose for the experiments performed in this study (Figure 1A).

### Effect of MPTP on Dopaminergic Neurons

One common discrepancy in PD literature is the correlation between tyrosine hydroxylase (TH) and dopaminergic neurons. Although a widely accepted proxy, TH is involved in the biosynthesis of epinephrine in addition to dopamine. Here we performed injections on Tg(*dat:eGFP*) zebrafish to analyze eGFP signal, providing a more direct assessment of dopaminergic degeneration as *dat* expression is localized specifically to dopaminergic neurons.

1-Methyl-4-phenyl-1,2,3,6-tetrahydropyridine was shown to impact dopaminergic neurons in three prominent regions within the zebrafish brain (Figure 1B); the olfactory bulbs (OBs; Figures 1C,D), telencephalon (Figures 1E,F) and periventricular pretectal nucleus (PPv; Figures 1G,H). The more immediate effects of MPTP were observed in the OBs with 2 and 38% reductions in eGFP+ cells following the first and second injections, respectively. In the OBs, this loss continued with each injection as MPTP-injected zebrafish resulted in losses of 32 and 47% following the third and fourth injections (Figure 1I). Likewise, MPTP-injected fish displayed reductions up to 41% in the telencephalon following the same injection regimen (Figure 1J). These effects were less severe within the PPv where a 25% loss of eGFP+ cells was observed following the final injection (data not shown).

To consolidate these findings at the genetic level, global brain transcripts for *dat* and *th1* were quantified using qRT-PCR. We found that both *dat* and *th1* expression levels decreased by 40 and 20%, respectively in MPTP-injected zebrafish (Figure 1K). Interestingly, we found 71 and 35% increases in *sox2* and *nestin* gene expression following the fourth injection point suggesting immediate regenerative activation following dopaminergic chemo-ablation (Figure 1K). To investigate potential pathways involved, we analyzed *shha* (*sonic hedgehog a*) expression, as *shha* has been identified as a key regulator of dopaminergic embryonic neurogenesis in zebrafish and other vertebrates (Hynes et al., 1995; Wullimann and Umeasalugo, 2020). We found a 29% increase in *shha* expression in MPTP-injected zebrafish; however, this increase did not reach statistical significance (Figure 1K). Our findings demonstrate the CVMI delivery of MPTP to induce considerable dopaminergic cell death and that there is a rapid neurogenic signaling response following the last injection.

### Effect of MPTP on Dopaminergic Mitochondria

**FIGURE 2:**
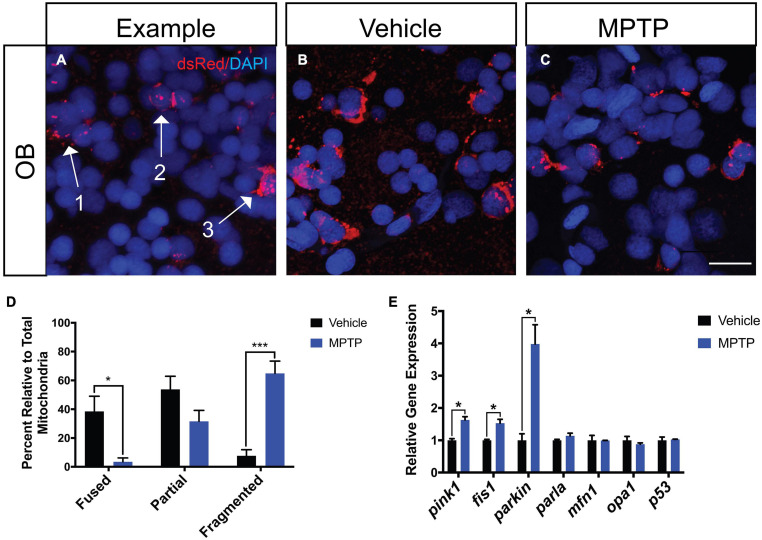
Effect of MPTP on mitochondrial fission-fusion dynamics in Tg(*dat:tom20 MLS:mCherry*) adult zebrafish. **(A)** Representative image depicting fragmented mitochondria (Arrow 1), partially fragmented mitochondria (Arrow 2), and fused mitochondria (Arrow 3). **(B,C)** Immunohistochemical labeling marked mCherry+ for mitochondrial visualization and nuclei with DAPI+ on coronal OB cryosections from vehicle and MPTP injected zebrafish. **(D)** Proportion of fused, partially fragmented and fragmented mitochondria (*n* = *4*). **(E)** Whole brain qRT-PCR analysis for mitochondrial and apoptotic genes; *mao-b*, *pink1*, *fis1*, *parkin*, *parla*, *mfn1*, *opa1*, and *p53* (*n* = 3 whole brains from each respective group). Scale bar = 10 μm. Bars represent the mean ± the SEM. *p < 0.05, ***p < 0.001.

To assess the effects of MPTP on dopaminergic mitochondrial morphology, we performed injections in Tg(*dat:tom20 MLS:mCherry*) zebrafish that label the mitochondria of dopaminergic neurons with mCherry (Noble et al., 2015). Following the last injection, cryosections were stained with dsRed, an antibody that recognizes mCherry, to visualize any potential disequilibrium between fission and fusion (Figure 2A). The OBs were selected for assessment due to their involvement in both sensory and motor function. We observed a significant increase in fragmented mitochondria of the MPTP-injected zebrafish relative to the PBS control (Figures 2B,C). Thus, 65% of all mitochondria analyzed in the MPTP-injected fish were fragmented, compared to only 8% in controls. Inversely, 4% of mitochondrial displayed a fused morphology in the MPTP-treated fish, compared to 39% in controls (Figure 2D).

To further support these findings, we analyzed genes associated with mitochondrial maintenance, structure and function. Most notable are increases observed in genes involved in mitophagy, where *pink1* is increased by 63% and *parkin* mRNA levels reach approximately four-times of those levels seen in controls. Consistent with the fragmented phenotype observed through IHC, *fis1* (*mitochondrial fission 1*) transcript levels were significantly increased by 53%, whereas *mfn1* (*mitofusion 1*) expression remained unaffected (Figure 2E). Moreover, we analyzed *opa1* (*optic dominant atrophy 1*) as it is involved in regulating mitochondrial cytochrome-c release and found expression to remain unchanged. The apoptotic marker *p53* was also analyzed and revealed that there was no effect of MPTP on this mechanism of cell death. Together these data suggest MPTP induces cell death in dopaminergic neurons via a mitophagy directed mechanism with mitochondrial fragmentation.

### Effect of MPTP on Zebrafish Behavior

To determine if the observed neurotoxicity translates into a locomotor or sensory phenotypes, zebrafish activities were monitored following every injection point and after a 2-week recovery period. The same individuals were analyzed throughout the experiment to delineate the effects of MPTP from individual variability within the population.

**FIGURE 3:**
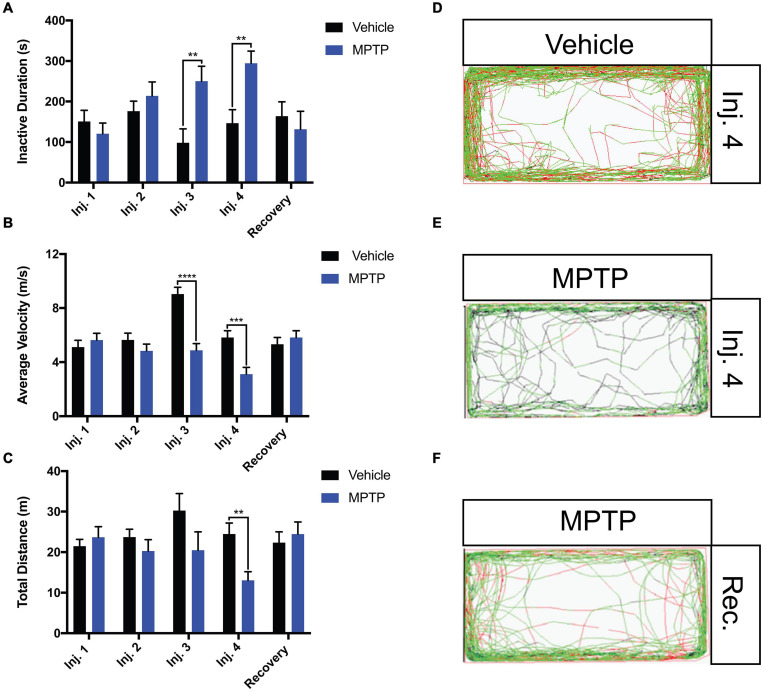
Impact of MPTP on adult zebrafish swimming behavior. Fish were acclimated to the apparatus for 15 min prior to behavioral analyses. Seven-minutes trials were performed. **(A–C)** Analysis of inactive durations (proxy for freezing bouts), average velocity and total distance for MPTP injected zebrafish and vehicle controls. Respective path images are as follows; injection 4 vehicle control **(D)**, injection 4 MPTP **(E),** and 2-week recovered MPTP treated zebrafish **(F)**. Red lines represent fast movement, green lines represent slow movement and black lines represent inactive movement below the slow threshold (*n* = 10 for all groups). Bars represent the mean ± the SEM. **p < 0.01, ***p < 0.001, and ****p < 0.0001.

The locomotion parameters that were examined included the total distance traveled, average velocity and freezing bout (inactivity) duration. MPTP-injected zebrafish elicited 46.7 and 47% reductions in total distance and average velocity relative to the controls following the fourth injection (Figures 3B,C). Inversely, freezing activity increased gradually with each injection and a 51% increase was observed following the fourth injection (Figure 3A). Notably, all swimming patterns returned to normal following a 2-week recovery representative path images shown in Figures 3D–F.

**FIGURE 4:**
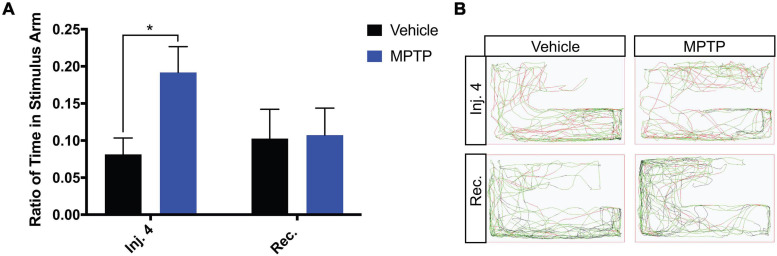
Impact of MPTP on olfactory response in adult zebrafish. **(A)** Ratio of time spent in the stimulus arm following the fourth injection and 2-week recovery. **(B)** Respective path images for MPTP injected zebrafish and vehicle controls after the fourth injection and a 2-week recovery. Red lines represent fast movement, green lines represent slow movement and black lines represent inactive movement below the slow threshold. Bars represent the mean ± the SEM. *p < 0.05.

Due to the substantial loss of dopaminergic neurons within the olfactory region, we sought to determine if this translates into any functional impairment of the olfactory response. To address this, we performed a repulsive stimulus test where cadaverine is administered to the arm of the apparatus the zebrafish is residing in. From quantifying the ratio of time spent in the stimulus arm, we found that cadaverine did not provoke a rapid repulsion response in MPTP-injected fish as observed in the controls. Control zebrafish spent 8% of the total time in the stimulus arm, whereas MPTP-injected zebrafish spent 19%, more than double the amount of the time in that arm (Figure 4A). Similarly to swimming activities, these effects were shown to return to normal 2-weeks post injection representative path images shown in Figure 4B. Together these data suggest that dopaminergic ablation via CVMI impairs the olfactory response and perturbs motor function.

## Discussion

### The Efficiency of CVMI for Dopaminergic Cell Death

Techniques to administer drugs will vary depending on the nature of the drug, the site of action and properties of the experimental animal. Popular administration methods include direct immersion, intraabdominal/IP and IM injections. However, each method comes with evident pitfalls. The largest downfall to IP injections is the primary route of absorption being through mesenteric vessels. Dosages are generally increased in this case to overcome the potential of the drug undergoing hepatic metabolism prior to systemic circulation (Abu-Hijleh et al., 1995; Turner et al., 2011). IM injections are commonly performed in mammalian models and are more difficult on zebrafish due to the reduced muscle mass and individual variability that poses the risk of differing drug absorption rates based on blood flow velocity (Manor and Sadeh, 1989; Dowd, 2017; Abbate et al., 2018). One must also be selective with the compound to be administered due to the potential for irritation and/or damage to the periphery nervous system that may result in paresis, paralysis or muscle necrosis (Rasmussen, 1978). Similarly, the direct immersion method of drug delivery makes it difficult to assess the exact quantity of drug being absorbed and can cause variability between treatments groups due to differing sizes, swimming patterns, and opercular movement speed (Cassar et al., 2020). The implementation of CVMI used in this study to administer MPTP did not result in any observable morphological abnormality nor systemic toxicity as observed using alternative methods (Bretaud et al., 2004; Kalyn et al., 2019).

1-Methyl-4-phenyl-1,2,3,6-tetrahydropyridine is commonly known as the “gold standard” in Parkinsonism studies due to the selective targeting of dopaminergic neurons. Alternative compounds may also act on other essential neuronal subtypes when injected in the absence of uptake inhibitors, such as 6-OHDA acting on both dopaminergic and epinephrinergic neurons (Fulceri et al., 2006; Bové and Perier, 2012). In zebrafish, the vast majority of MPTP studies were performed on embryos and/or larvae. However, these studies have laid the groundwork and outlined that MPTP affects ascending TH + diencephalic (5, 6, and 11) and pretectal clusters (7) more severely than others, while also decreasing *dat* and *th1* transcript and neurotransmitter levels (Sallinen et al., 2009; Kalyn et al., 2019). Moreover, MPTP and MPP+ have been shown to translate into motor perturbing phenotypes in 3, 5, 6, and 7 dpf zebrafish (Lam et al., 2005; Sallinen et al., 2009; Díaz-Casado et al., 2016; Kalyn et al., 2019; Zhao et al., 2020). Larval studies have also identified a variety of compounds that can be used in a pre-treatment exposure to mitigate some degree of MPTP induced dopaminergic neurodegeneration. A few of these compounds include deprenyl, melatonin and rosmarinic acid which act to alleviate stress through MAO-B inhibition, anti-oxidation and/or anti-inflammation (Parng et al., 2006; Sallinen et al., 2009; Díaz-Casado et al., 2016; Zhao et al., 2020). In adults, reports primarily consist of behavioral assessment without much examination of dopaminergic neurons and their mitochondria (Flinn et al., 2008; Selvaraj et al., 2019). However of the few that have investigated dopaminergic health, Anichtchik et al. (2004) have shown that a systemic injection of MPTP resulted in decreases in dopamine and noradenaline concentrations in the brain without any effect on TH immunoreactivity, TH co-localization with TUNEL or any increase in caspase-3 activity. Moreover, Bretaud et al. (2004) did not observe any dopaminergic neuron ablation following IP injections of MPTP, rotenone, or paraquat (other toxins implicated in PD studies). Here we report that CVMI was a successful method to deliver MPTP into the adult zebrafish brain through the observed increases in dopaminergic cell death within the telencephalic and olfactory regions of the adult brain, as well as, the spatial disorganization and slight reduction of dopaminergic neurons in the PPv.

To assess the impact on dopaminergic neurons and further support our findings, *th1*, *mao-b*, and *dat* gene expression profiles were evaluated. Expression of *th1* was analyzed in contrast to *th2* as *th2* is exclusively expressed in non-mammalian vertebrates and present in only four hypothalamic neuronal clusters within the hypothalamic and posterior tubercular regions of the brain (Barrios et al., 2020). The increased MAO-B mRNA expression observed suggests the continuous conversion of MPTP into its active toxic form MPP+ even following the fourth injection point. MPP+ then exerts its effects on cells expressing *th1* and *dat*, as shown through decreases in respective gene mRNA levels. The >50% *dat* expression remaining can be explained by the remaining dopaminergic neurons expressing *dat* as a means to recycle synaptic dopamine into the cytosol of dopaminergic neurons (Shimizu and Prasad, 1991; Giros et al., 1992; Sulzer et al., 2016; Merhi et al., 2021). Although we cannot rule out the possibility of a transient downregulation in *dat*/*th1* expression that may contribute to the functional impairment and recovery of zebrafish behavior, the effect of MPTP on *tom20* expressing dopaminergic neurons combined with the eGFP data are suggestive of neurodegeneration occurring.

Interestingly, increases in expression for neural proliferative genes and *shha* were observed immediately following the last injection of MPTP. Shh is a main signaling morphogen in the ventralization of vertebrate neural tubes (Hynes et al., 1995; Wang et al., 2009; Carballo et al., 2018). Particularly in zebrafish, *shha* expressing cells have been shown to give rise to preglomerular complex and posterior tubercular dopaminergic neurons (Wullimann and Umeasalugo, 2020). Given the activity of Shh in embryonic dopaminergic neurogenesis and the increase in mRNA transcript levels observed in the present study, further analysis of Shh is warranted to determine its precise role in adult dopaminergic neurogenesis following ablation.

### Mitochondrial Response to MPTP in Dopaminergic Neurons

Mitochondrial dysfunction is a hallmark of neurodegeneration in PD patients. There are various processes (fission, biogenesis, and mitophagy) that regulate mitochondrial quality within all cell types (Kobayashi et al., 2020). Fission is a mechanism that activates mitophagy and plays an adaptive role in the clearance of damaged mitochondria in response to oxidative stress or injury. Mitophagy is the process by which fragmented mitochondria are sequestered and degraded by lysosomes (Ding and Yin, 2012). Regarding biogenesis, it is well characterized that mitochondria provide energy to the cell through two dominant pathways: glycolysis and oxidative phosphorylation (Westermann, 2012). The variations in energetic supply or metabolic cues can also influence mitochondrial morphology (Yu and Pekkurnaz, 2018). Glycolytic mitochondria generally display a fragmented appearance with fission contributing to mitochondrial proliferation, while mitochondria undergoing oxidative phosphorylation display a more fused conformation (Rossignol et al., 2004; Westermann, 2012; Youle and van der Bliek, 2012; Seo et al., 2018). Dependent on the energetic requirements, highly active cells commonly rely on oxidative phosphorylation (Westermann, 2012). Dopaminergic neurons fall into this category.

Many studies have outlined the mechanism of MPTP mediated cell death via the inhibition of the ETC (Przedborski et al., 2000, 2004; Keane et al., 2011). However, a knowledge gap remains in visualizing these effects on mitochondrial conformational changes in the dopaminergic neurons of an adult zebrafish brain. Through performing injections on Tg(*dat:tom20 MLS:mCherry*) zebrafish, we were able to observe the direct consequences of MPTP on mitochondrial morphology. Twenty-four hours following the fourth injection, MPTP-injected zebrafish exhibited significant fragmentation in dopaminergic neurons. Consistent with the results indicative of mitochondrial fission, there are significant increases in genes associated with this phenomenon: *fis1* (*mitochondrial fission protein 1*), *parkin* and *pink1 PTEN-induced kinase 1*). The Parkin/Pink1 pathway is active in all mitochondria of healthy cells to expel dysfunctional or damaged mitochondria through mitophagy. Thus, damaged mitochondria in highly energetic cells will display a fragmented morphology. Addressing this phenomenon, Park et al. (2019) found increased numbers of abnormal mitochondria within dopaminergic neurons substantia nigra in macaques following MPTP exposure. However, this study outlined the mechanism of fission through Drp1 and Fis1 activation and not necessarily the subsequent cell death. Taken together with the decrease in eGFP+ expressing cells in our results, it is possible that the remaining dopaminergic neurons are degenerating via a mitophagy dominant mechanism inducing mitochondrial fission and neuronal death.

In addition to fragmentation as a response of oxidative stress and given that cellular bioenergetic demand can influence mitochondrial morphology, we cannot disregard that some dopaminergic neurons may be undergoing metabolic reprogramming from oxidative phosphorylation to aerobic glycolysis following MPTP injections. Emerging studies have shown that similar modifications in mitochondrial networking and structure occur as a mechanism to bioenergetically adapt to stress and/or inflammation (Baker et al., 2014). Nair et al. tested this hypothesis by inducing fission and energetic reversion to glycolysis through lipopolysaccharide (LPS) exposure in microglia. They subsequently found that a Drp1 protein inhibitor, Mdivi-1, attenuates reactive oxygen species (ROS) production, fragmentation and pro-inflammatory responses following LPS treatment *in vivo* and *in vitro* (Nair et al., 2019). Further analysis of genes and/or proteins involved in the present study must be done to support this possibility of dopaminergic mitochondrial metabolic reprogramming. One mechanism of interest was suggested by Zheng et al. (2016) who observed a loss of expression from lactate dehydrogenase and hexokinase, in parallel to the conversion in pyruvate kinase splicing from PKM2 to PKM1, that efficiently labels the transition from aerobic glycolysis in progenitor cells to oxidative phosphorylation in maturing neurons.

### Behavioral Consequence Following MPTP Injections

The dopaminergic system is critical to several biological functions that include reward, pleasure, movement, and learning. Incidentally, the consequence of ablating dopamine producing cells can result in severe behavioral phenotypes. Microinjections of MPTP are shown to affect the dopaminergic projectome that modulates movement through observed impaired swimming parameters. MPTP-injected fish exhibited increased freezing bout duration, concurrent with decreases in both total distance and average velocity relative to the respective controls, resembling bradykinetic and dyskinetic symptoms (Vaz et al., 2018). MPTP-injected zebrafish displayed impaired olfactory response to the repulsive stimulus cadaverine. With the telencephalic and olfactory regions being two epicenters, in the zebrafish brain, that modulate behavior, this impact can be explained by the drastic decreases in eGFP+ cells residing within the OBs and telencephalon in MPTP-injected zebrafish. These effects are greater than those observed in our group’s previous work from Godoy et al. (2020), that used a chemogenetic approach to ablate dopaminergic neurons. The phenotypes observed in this study were transient as all motor and sensory functions were restored following a 2-week recovery period. This capacity for regeneration alone makes the zebrafish advantageous in neurodegenerative studies, however, this also poses a hindrance toward long-term therapeutic studies as it may be difficult to delineate any restorative effects between the therapeutic compound administered and the natural regeneration that occurs. Therapeutic studies using this model commonly treat the zebrafish prior to neuronal insult to evaluate the efficiency of varying neuroprotective compounds. Given the 2-week transiency of sensorimotor phenotypes, it would be beneficial to implement a short-term regenerative study following ablation to identify possible neurogenic drivers of dopaminergic neurons in adult zebrafish to gain a better temporal understanding of behavioral restoration. As previously mentioned, identifying drivers, such as Shh, may serve as a foundation for gene-therapy application in higher vertebrate models.

## Conclusion

Literature surrounding PD suffers from discrepancies among the degree of dopaminergic ablation and behavioral phenotypes resulting from neurotoxin treatments. Here, we demonstrated that CVMI delivery of MPTP induces substantial dopaminergic neuron death within the telencephalon and olfactory regions of the adult zebrafish brain. This reduction in dopaminergic neurons resulted in transient sensory and motor phenotypes. Moreover, using the Tg(*dat:tom20 MLS:mCherry*) zebrafish, we were able to observe a significant increase in the proportion of fragmented mitochondria in MPTP-injected zebrafish. Supporting gene expression evidence suggests that the dopaminergic cell death is activated via a mitophagy mechanism that results in increased fission and a more fragmented mitochondrial morphology. Taken together this study will ameliorate our understanding of chemically induced mitochondrial dysfunction in the neurodegeneration of dopaminergic neurons and other PD pathologies.

## Data Availability Statement

The original contributions presented in the study are included in the article/supplementary material, further inquiries can be directed to the corresponding author.

## Ethics Statement

The animal study was reviewed and approved by the University of Ottawa Animal Care Committee.

## Author Contributions

MK and ME: conceptualization, methodology, validation, and writing – review and editing. MK: data curation, formal analysis, supervision, visualization, and writing – original draft. Both authors have read and agreed to the published version of the manuscript.

## Conflict of Interest

The authors declare that the research was conducted in the absence of any commercial or financial relationships that could be construed as a potential conflict of interest.

## Publisher’s Note

All claims expressed in this article are solely those of the authors and do not necessarily represent those of their affiliated organizations, or those of the publisher, the editors and the reviewers. Any product that may be evaluated in this article, or claim that may be made by its manufacturer, is not guaranteed or endorsed by the publisher.
